# Heterologous Expression of *Salvia miltiorrhiza* MicroRNA408 Enhances Tolerance to Salt Stress in *Nicotiana benthamiana*

**DOI:** 10.3390/ijms19123985

**Published:** 2018-12-11

**Authors:** Xiaorong Guo, Junfeng Niu, Xiaoyan Cao

**Affiliations:** National Engineering Laboratory for Resource Development of Endangered Crude Drugs in Northwest of China, Key Laboratory of the Ministry of Education for Medicinal Resources and Natural Pharmaceutical Chemistry, Shanxi Normal University, Xi’an 710062, China; guoxiaorong@snnu.edu.cn

**Keywords:** abiotic stress, antioxidant enzyme, miR408, salt tolerance, *Salvia miltiorrhiza*

## Abstract

MicroRNAs (miRNAs) are a class of endogenous small RNAs that regulate the expression of target genes post-transcriptionally; they are known to play major roles in development and responses to abiotic stress. MicroRNA408 (miR408) is a conserved small RNA in plants; it was reported that miR408 genes were involved in abiotic stress in *Arabidopsis*. However, miR408 in *Salvia miltiorrhiza* has been rarely investigated. In this study, we cloned *Sm-MIR408*, the miR408 precursor sequence, and its promoter sequence from *S. miltiorrhiza* and the role in tolerance to salt stress is described. The effects of salt stress on miR408 expression were studied by using β-glucuronidase (GUS) staining. Our data indicated that transgenic tobacco overexpressing *Sm-MIR408* promoted seed germination and reduced the accumulation of reactive oxygen species under salt stress. Transcript levels of antioxidative genes, i.e., *NbSOD*, *NbPOD*, and *NbCAT*, and their enzyme activities increased in salinity-stressed transgenic tobacco plants, suggesting a better antioxidant system to cope the oxidative damage caused by salinity stress. Taken together, these findings indicated that miR408 functions in positive responses to salt tolerance in tobacco.

## 1. Introduction

Abiotic stresses such as salinity, drought, cold, and extreme temperature greatly affect the growth and development of plants. In order to cope with these challenges, plants have evolved complex molecular mechanisms to deal with unfavorable environments and to reduce the damage as much as possible, resulting in adaptive responses through physiological and morphological changes [1,2]. Increasing salinization of soil is one of the main factors influencing metabolic imbalance, excess accumulation of reactive oxygen species (ROS), reduction in photosynthetic performance, and nutrient absorption reduction [3,4]. Plants respond to the unfavorable environment and minimize possible damage by readjusting their physiological and biochemical levels. For example, ROS (H_2_O_2_, –OH) produced by aerobic metabolism can destroy cellular homeostasis and lead to cell death [5], and the ROS-scavenging system, which is mainly assisted by enzymatic systems, can reduce the impact of ROS on plants. Due to the unavoidable effects of abiotic stress on plants, it is critical to develop an effective and reliable procedure to mitigate the effects.

MicroRNAs (miRNAs) are a class of small non-coding RNAs 20–24 nucleotides in length, and they are derived from stem-loop precursors that repress target gene expression at the post-transcriptional level [6]. After integration into the RNA-induced silencing complex (RISC), miRNAs can form near-perfect pairs with their mRNA targets, based on sequence complementarity, and direct cleave the target genes or translational repression to play its biological function. As a new gene regulator, miRNAs have a similar function with the transcription factors [7,8] which regulate development, growth and stress response in plants [9]. Various studies have demonstrated that miRNAs regulate the development and morphogenesis of leaves [10,11,12] and participate in the development of flowers [13,14,15]. Additionally, miRNAs respond to biotic and abiotic stresses like drought, salt, cold, and osmotic stress. For instance, drought stress induces miR159 [16] and miR319 [17] to accumulate in *Arabidopsis*, and the expression of miR156, miR159, and miR396 increased significantly under the condition of salt stress. Osmotic stress reduces the expression of miR398 and targets *CSD1* and *CSD2* that positively regulate oxidative stress tolerance in *Arabidopsis*, with miR398 as a positive regulator of oxidative stress responses [18].

MiR408 is a small RNA of 21 nucleotides, which was first discovered in *Arabidopsis*. At present, miR408 family comprises 56 members annotated in more than 30 plant species in miRBase database. The target genes of miR408 are blue copper protein members divided into two categories, including copper-binding proteins and laccase [19,20]. Targets of miR408 maintain copper homeostasis, and copper plays an important role in plants involved in photosynthesis, mitochondrial respiration, and lignification of the cell wall. Furthermore, the expression of miR408 is significantly influenced by various growth and environmental conditions. For example, miR408 is up-regulated in response to drought in *Medicago truncatula* [21] and *Arabidopsis* [22], while it is reduced in *Oryza sativa* [23] and *Prunus persica* [24]. Recent research has shown that miR408 is an important component of the HY5-SPL7 gene network that mediates the coordinated response to light and copper [25]. In *Arabidopsis*, overexpression of miR408 results in increased resistance to salinity, cold, and oxidative stress, but enhanced sensitivity to drought and osmotic stress [26]. Meanwhile, transgenic chickpea overexpressing miR408 showed increased drought tolerance [27]. Together, these results reveal that miR408 is involved in abiotic stress responses in plants, and it plays different roles in different species.

*Salvia miltiorrhiza* is one of the most commonly investigated medicinal plants, and its root is a traditional Chinese medicine for treating thoracic obstruction and heartache, amenorrhea, dysmenorrheal, and upset insomnia [28]. Now, it has been intensively studied for its protective role against cardiovascular diseases [29]. Although a few documents about microRNAs in *S. miltiorrhiza* have been reported [30,31], there is no report to date about functional study of microRNA from this species. In our previous work, we cloned a 366 bp miR408 precursor sequence from *S. miltiorrhiza* and named it *Sm-MIR408* [32]. Bioinformatics analysis showed that *Sm-MIR408* can form a stable stem loop structure and it had a higher homology with tobacco and potato [31]. Tobacco belongs to the genus *Nicotiana* in the family Solanaceae, and is also readily transformable with a relatively short life cycle, which makes it a model plant for genetic studies.

To characterize the function of *Sm-MIR408*, we analyzed the functions of the 723-bp promoter region upstream of the predicted transcription initiation site of the *Sm-MIR408*-driven gene for β-glucuronidase (GUS), and *Sm-MIR408* overexpressed in transgenic *Nicotiana benthamiana*, respectively. Our results showed that the expression of *Sm-MIR408* is induced by salt treatment. Furthermore, overexpression of *Sm-MIR408* in tobacco promoted seed germination under salt stress, and increased resistance to salt by activating the ROS-scavenging system. To our knowledge, this is the first report about a functional study of *S. miltiorrhiza* miRNA. Our data support that miR408 might serve as a potential target for genetic manipulations to engineer salt stress tolerance in diverse plants.

## 2. Results

### 2.1. Sm-MIR408 Is Induced by Copper Deficiency and Salt Treatment

**Figure 1:**
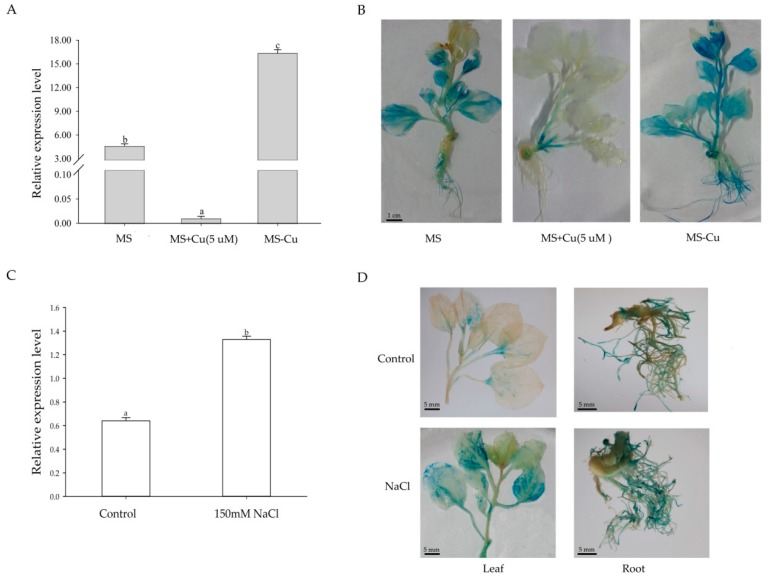
Transcription of *Sm-MIR408* is induced under deficient copper conditions or NaCl treatment. (**A**) Expression level of *Sm-MIR408* when two-week-old *S. miltiorrhiza* seedlings were transferred to standard Murashige and Skoog (MS) medium (0.1 μM copper), MS medium deficient in copper (0 μM, MS−Cu) and sufficient copper (5 μM, MS+Cu) for 7 days. (**B**) β-glucuronidase (GUS) staining results when one-week-old transgenic *Nicotiana benthamiana* expressing *MIR408pro::GUS* were transferred to MS, MS−Cu and MS+Cu for three weeks. (**C**) Expression levels of *Sm-MIR408* in one-month-old *Salvia miltiorrhiza* seedlings treated with 150 mM NaCl for 24 h. (**D**) The GUS staining in the transgenic *Nicotiana benthamiana* expressing *MIR408pro::GUS* after treatment with 150 mM NaCl. For RT-qPCR, all data are means of three biological replicates, with error bars indicating SD; significant differences were determined using Duncan’s multiple range test (indicated by different letters at *p* < 0.05).

It was observed in *Arabidopsis* that the abundance of miR408 is responsive to copper supply in the environment. The miR408 level was low with standard Murashige and Skoog (MS) media (with a copper concentration of 0.1 μm), but it increases markedly upon copper starvation [33]. Supplementing MS with 5 μm copper effectively prevents the accumulation of miR408 [34]. Since miR408 is highly conserved in land plants, we speculate that *Sm-MIR408* in *S. miltiorrhiza* probably responds to copper supply in a similar expression pattern to that in *Arabidopsis*. Here, we analyzed its expression when *S. miltiorrhiza* was grown on standard MS medium (0.1 μM copper), MS medium-deficient copper (0 μM, MS−Cu) and sufficient copper (5 μM, MS+Cu) conditions. Real-time quantitative PCR (RT-qPCR) results showed that the expression of *Sm-MIR408* was strongly influenced by copper availability (Figure 1A). To further verify the expression of *Sm-MIR408* in response to copper, transgenic tobacco expressing *MIR408pro::GUS* were cultured on MS, MS−Cu and MS+Cu medium. GUS staining revealed that the GUS signal was increased in transgenic tobacco grown on MS−Cu medium, and greatly decreased on MS+Cu medium, when compared with that grown on standard MS medium (Figure 1B). Our data indicated that transcription of *Sm-MIR408* was strongly induced by copper deficiency, which was consistent with the results in *Arabidopsis* [33,34].

Since miR408 is involved in abiotic stress responses in plants [26,27], we further analyzed its expression under salt stress conditions. Real-time quantitative PCR (RT-qPCR) results showed that salt treatment significantly enhanced the expression of *Sm-MIR408* in the seedlings of one-month-old *S. miltiorrhiza* (Figure 1C). Then, one-month-old T2 generation transgenic tobacco expressing *MIR408pro::GUS* were used to verify the expression of *Sm-MIR408* in response to salt treatment. GUS staining revealed that the GUS signal was increased in both the leaves and roots of transgenic tobacco treated with 150 mM NaCl for 24 h, compared to that of the control (Figure 1D). Altogether, these data suggest that the expression of *Sm-MIR408* is up-regulated under salt stress condition.

### 2.2. Heterologous Expression of Sm-MIR408 in Nicotiana benthamiana

**Figure 2:**
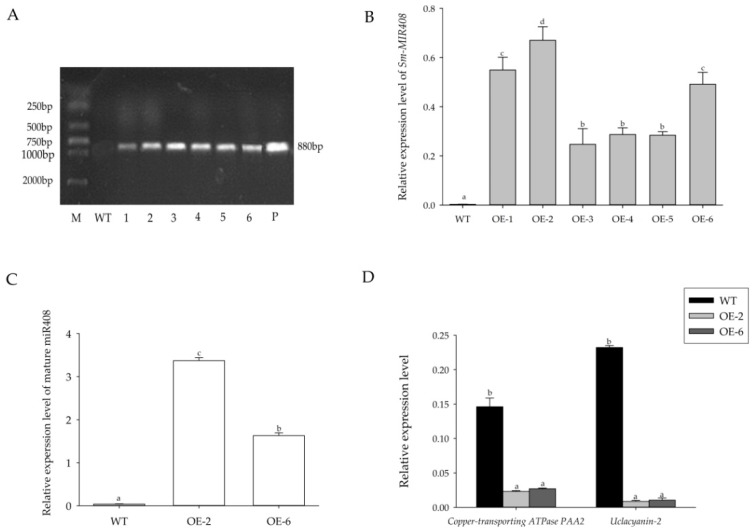
Detection of Sm-miR408 overexpressing (OE) transgenic lines. (**A**) PCR amplification product of 35S promoter from transgenic *Nicotiana benthamiana* genomic DNA (gDNA). Lanes: M, DL2000 DNA marker; WT, wild-type plants as negative control; 1-6, different transgenic lines; P, plasmids as positive control. (**B**,**C**) Relative expression levels of *Sm-MIR408* (**B**) and mature miR408 (**C**) in transgenic lines and WT. (**D**) Relative expression levels of *copper-transporting ATPase PAA2* and *uclacyanin-2* in transgenic lines and WT. The data represent means ± SD of three independent experiments. Significant differences were determined using Duncan’s multiple range test (indicated by different letters at *p* < 0.05).

To evaluate the role of miR408 in the plant adaption to salt stress, we generated a *Sm-MIR408* overexpression construct *35S::MIR408* driven by the cauliflower mosaic virus 35S (*CaMV35S*) promoter, which was introduced into the genome of tobacco through *Agrobacterium tumefaciens*-mediated transformation. To select positive transgenic plants overexpressing (OE) *Sm-MIR408*, we amplified the *CaMV35S* promoter by PCR with the genomic DNA of regenerated plants, and identified a total of six independent transgenic lines (Figure 2A). To verify the expression of *Sm-MIR408* in transgenic tobacco, we conducted RT-qPCR to analyze the transcriptional levels of *Sm-MIR408* in the transgenic lines. As demonstrated in Figure 2B, the transgenic lines showed different transcript abundances, while the transcripts of *Sm-MIR408* was not detected in the wild-type (WT) control. To determine whether *Sm-MIR408* can be successfully processed into mature miRNA, we conducted stem-loop RT-qPCR analyses and found that the expression level of mature miR408 in transgenic lines were significantly higher than that in the WT (Figure 2C), suggesting that the transcripts of *Sm-MIR408* is properly processed into mature miR408.

Based on the expression level of miR408, two transgenic lines, OE2 and OE6, were used in the following experiments. Since the targets of Nb-miR408 were *copper-transporting ATPase PAA2* and *uclacyanin-2* in *N. benthamiana* [35], we further analyzed their expression levels in the *Sm-MIR408*-overexpressing transgenic lines and WT by RT-qPCR. Our results indicated that the expression levels of *copper-transporting ATPase PAA2* and *uclacyanin-2* in transgenic lines were significantly lower than those in WT (Figure 2D).

### 2.3. Sm-miR408 Confers Abiotic Stress Tolerance to Transgenic Plants

**Figure 3:**
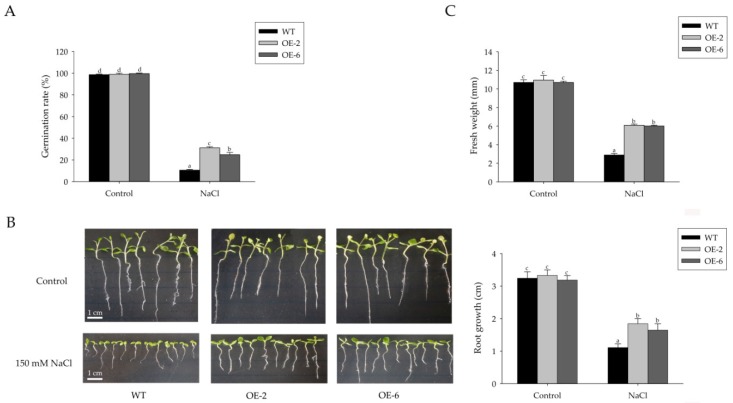
Salt stress tolerance assays of transgenic *Nicotiana benthamiana*. (**A**) Germination rates of wild type (WT) and miR408-overexpressing tobacco (OE2 and OE6) on MS medium containing 150 mM NaCl for 7 days. (**B**,**C**) The seedling phenotype (**B**) and statistical analysis of root length and fresh weight (**C**) when the seeds of transgenic lines (OE2 and OE6) and WT tobacco were cultured on MS medium and MS medium containing 150 mM NaCl for 21 days The error bars represent standard deviations of the mean measurements. Significant differences were determined using Duncan’s multiple range test (indicated by different letters at *p* < 0.05).

To characterize the role that miR408 plays in abiotic stress tolerance, T1 transgenic lines OE2 and OE6 were selected for function analysis. The seeds of WT and transgenic lines were germinated on MS plates containing 150 mM NaCl to investigate seed germination and early seedling growth. Under normal growth condition, no significant difference was found in seed germination rates between WT and transgenic lines (Figure 3A). However, transgenic lines exhibited significantly higher germination rates than that of the WT on MS plates with 150 mM NaCl (Figure 3A). We further observed the growth phenotype of the transgenic lines and WT seedlings. As shown in Figure 3B,C, the transgenic lines and WT seedlings showed no significant phenotypic differences under normal conditions, while OE2 and OE6 exhibited improved root growth and significantly higher fresh weights than WT after cultivating for 21 days with salt treatment. The results indicated that overexpression of miR408 enhanced the tolerance to salt in tobacco.

### 2.4. Overexpression of Sm-miR408 Reduced ROS Accumulation under Salt Stress

**Figure 4:**
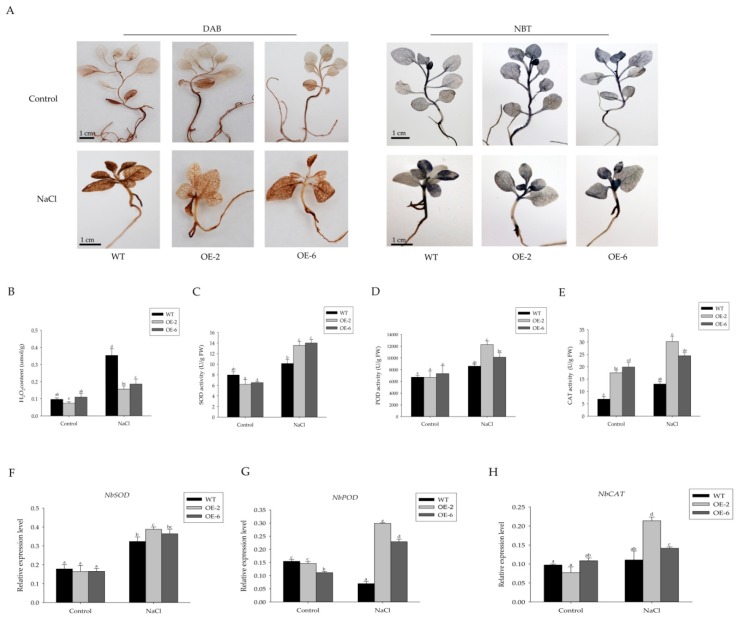
Contents of reactive oxygen species (ROS) in wild type (WT) and miR408-overexpressing *Nicotiana benthamiana*. (**A**) 3,3-Diaminobenzidine (DAB) and nitroblue tetrazolium (NBT) staining when three-week-old seedlings were treated with 150 mM NaCl for 10 days. The strength of color showed the concentration of H_2_O_2_ and O_2_^−^. (**B**) Contents of H_2_O_2_ in tobacco with or without salt treatment. (**C**–**E**) The activities of SOD (**C**), POD (**D**), and CAT (**E**) in WT and transgenic lines when three-week-old seedlings were treated with 150 mM NaCl for 10 days. (**F**–**H**) Expression of ROS-responsive genes *NbSOD* (**F**), *NbPOD* (**G**), and *NbCAT* (**H**), determined via RT-qPCR. The data represent means ± SD of three independent experiments. Significant differences were determined using Duncan’s multiple range test (indicated by different letters at *p* < 0.05).

The abiotic stress leads to the generation of ROS, and the plants cope with excess ROS through its enhanced antioxidant defense system. Therefore, we examined the accumulation of hydrogen peroxide (H_2_O_2_) and superoxide anion radical (O_2_^−^) in the WT and transgenic seedlings under salt stress by histochemical staining with 3,3′-diaminobenzidine (DAB) and photometric nitro blue tetrazolium (NBT). As shown in Figure 4A, under normal growth conditions, there were no differences in H_2_O_2_ and O_2_^−^ between WT and transgenic lines. Although brown-colored polymeric oxidation products were visualized in all of the salt-stressed plants, transgenic plants exhibited lower accumulations of H_2_O_2_ than WT plants (Figure 4B).

Enzyme antioxidants play an important role in scavenging reactive oxygen species. Therefore, we assessed the activities of peroxidase (POD), superoxide dismutase (SOD), and catalase (CAT) in transgenic lines and WT. As shown in Figure 4C–E, the activities of three antioxidant enzyme were all up-regulated in all the seedlings with salt treatment, while significantly higher in the transgenic plants than those in WT. The higher activity of SOD, POD, and CAT resulted in lower levels of accumulation of O_2_^−^ and H_2_O_2_ in transgenic tobacco. RT-qPCR was performed to detect the expression levels of ROS-related genes in the WT and transgenic lines with or without treatment. The results showed that the expression levels of *NbPOD*, *NbCAT*, and *NbSOD* in transgenic lines were significantly higher than those in the WT under salt stress (Figure 4F–H). These results suggested that overexpression of *Sm-MIR408* reduced ROS accumulation by activating ROS-related genes and enhancing SOD, POD, and CAT activities under salt stress in tobacco.

## 3. Discussion

MicroRNAs (miRNAs) are a class of small non-coding RNAs 20–24 nucleotides in length. Apart from their roles in development, the roles that some of their members play in the complex stress response network have been gradually recognized. For example, overexpressing Osa-miR528 enhances tolerance to nitrogen starvation in creeping bentgrass [36]. Overexpression of miR172a and miR172b in *Solanum lycopersicum* enhances resistance to *Phytophthora infestans* [37]. Heterologous expression of a rice miR395 gene in *Nicotiana tabacum* impairs sulfate homeostasis [38]. Transgenic *Agrostis stolonifera* overexpressing *Osa-miR393a* improved multiple stress tolerance, including drought, salt and heat [39]. Transgenic barley that overexpressed hvu-miRX showed drought tolerance [40]. The increasing number of functional studies on plant miRNAs have demonstrated that they are promising candidates for enhancing multiple stress tolerance in plants.

MiR408 is a highly conserved miRNA, and it plays significant roles in plant growth and development. In *Brassica napus*, the upregulation of miR408 restricted silique development due to inorganic phosphate/copper deficiency [41]. MiR408 regulates vegetative development in *Arabidopsis* and heading time in wheat [34,42]. The constitutive expression of miR408 can improve photosynthesis, growth, biomass, and seed yield in some plants [43,44,45]. Recent studies have shown that abiotic stresses induce the aberrant expression of miR408. MiR408 is a typical of miRNAs that respond to the abiotic stress differently depending on the plant species. For example, drought treatment up-regulated miR408 expression in *Arabidopsis* [22] and *Medicago truncatula* [21], but down-regulated the expression of miR408 in *Oryza sativa* [23], *Prunus persica* [24], and cotton [46]. The expression of miR408 was up-regulated in response to salinity treatment in *Arabidopsis* [22] and cotton [45], while it was significantly down-regulated in response to salinity stress in rice [47] and radish [48]. Here, we investigated the expression of *Sm-MIR408* in response to salt stress. RT-qPCR and GUS staining revealed that miR408 was up-regulated by salinity treatments in *S. miltiorrhiza* (Figure 1), which was consistent with the expression pattern in *Arabidopsis* and cotton.

Although the function of miR408 as a key post-transcriptional regulator of gene expression in plant development is well established, its functional response to the environment is very limited. By now, only two documents about the function of miR408 response to abiotic stress have been reported [24,26]. In *Arabidopsis*, overexpression of miR408 enhances its tolerance to salinity, cold, and oxidative stress, but improved sensitivity to drought and osmotic stress [24]. Transgenic chickpea overexpressing the miR408 increased drought tolerance [26]. These studies hinted that miR408 plays important roles in abiotic stress, but it probably plays the opposite function in different species. Salt stress is one of the main environmental stresses that have a severe effect on the quality and yield of crops. Base on the result that miR408 was up-regulated by salinity treatments in *S. miltiorrhiza* (Figure 1C,D), the function of the miR408 response to salt stress was carried out in the present study. Transgenic tobacco overexpressing *Sm-MIR408* showed higher germination rates and lower growth inhibition than WT under salt stress conditions (Figure 3). Our results indicated that a high expression of miR408 improved tolerance to salinity in tobacco, which agreed with the function of miR408 in *Arabidopsis* [24].

ROS are important signaling molecules in the regulation of many of biological processes. The accumulation of ROS was enhanced in tissues when plants suffer abiotic stresses. Many studies have revealed that the capacity of ROS scavenging was associated with plant tolerance to abiotic stresses [49,50,51,52]. In the present study, all the plants accumulated higher ROS levels under salt stress conditions, while transgenic plants exhibited lower accumulation than WT (Figure 4B). Due to the physical and chemical toxicity of high ROS levels, the ROS-scavenging enzymes, including SOD, POD, and CAT, are activated in plant cells in response to oxidative damage [53]. The low content of ROS is related to the high activity of those enzymes [54]. So, we tested the activity of SOD, POD, and CAT in plants. As shown in Figure 4C–E, the activities of SOD, POD, and CAT were higher in transgenic lines than those in the WT plants under salt stress. However, differences could be observed between the two independent transgenic lines. The relative increase in the CAT and POD activity was higher in OE2 compared to OE6. We speculated that higher expression level of miR408 in OE2 is correlated with higher increase of enzyme activity in that line, supporting its involvement in reducing the accumulation of ROS during salinity stress.

MiRNAs play their roles by regulating the expression of target genes. In *Populus euphratica*, the target *PeNAC* genes of peu-miR164 were involved in abiotic stress responses [55]. MiR408 is negatively regulated by the availability of copper in *Arabidopsis* and target genes encoding several copper transporters and a copper chaperone, indicating its central role in the response to copper deficiency [33,34]. Here, RT-qPCR and GUS staining results indicated that *Sm-MIR408* in *S. miltiorrhiza* was strongly induced by copper deficiency, and it was efficiently inhibited by sufficient copper (Figure 1A,B). MiR408 targets genes encoding Copper-transporting ATPase PAA2 and Uclacyanin-2 (UCL-2) in *N. benthamiana* [35]. Copper-transporting ATPase PAA2 is a copper-transporter that supplies Cu to plastocyanin (PC), an essential protein for photosynthetic activity in higher plants [56]. Uclacyanin-2 belongs to phytocyanin family, which are blue copper proteins and function as electron transporters [44]. Both targets are related to copper, which is a cofactor of various enzymes, and plays roles in photosynthesis and respiration electron transfer, oxidative stress protection, ethylene perception, and cell wall metabolism [44]. Also, 14 *UCLs* were regulated under the treatments of abiotic stresses [57]. It was hypothesized that the release of copper from non-essential cuproproteins can act as copper supply buffer [58]. Such a copper release and supply mechanism could be activated during stress responses and supply the copper required for proteins involved in stress responses [26]. We hypothesize that constitutive expression of miR408 resulted in reduced levels of the non-essential cuproprotein Uclacyanin-2, leading to increased availability of copper to those cuproproteins that are required to cope with the stresses, such as the Cu/Zn SODs. The connection between the targets of miR408 and antioxidative system or genes involved in abiotic stress response is yet to be understood with greater details.

## 4. Materials and Methods

### 4.1. Plant Material and Growth Conditions

All the plants (including the wild-type *S. miltiorrhiza*, wild-type *N. benthamiana*, transgenic *S. miltiorrhiza* and transgenic *N. benthamiana*) were grown in a greenhouse under standard growth conditions of light and temperature (22 °C, light intensity 100 μmol m^−2^·s^−1^, 16/8 h light/dark cycle) with 60% relative humidity.

The seeds of transgenic tobacco expressing *35S::MIR408* and WT were cultured vertically on MS medium and MS medium containing 150 mM NaCl and germination rate was recorded after 7 days. The root length and fresh weight were measured when the seeds were cultured for 21 days under normal and salt stress conditions. Three-week-old transgenic seedlings and WT grown under normal condition were transferred to MS medium or MS medium supplemented with 150 mM NaCl for 10 days, and harvested for the histochemical detection of ROS, RT-qPCR, the determination of H_2_O_2_ content, and measurements of SOD, POD, and CAT activity. In the experiment, at least 10 seedlings were used for each treatment. All the experiments were performed with three biological replicates.

### 4.2. Construction of Plant Expression Vector and Tobacco Transformation

Amplification of 366 bp miR408 precursor sequences was made from the genomic DNA of *S. miltiorrhiza* using the primers MIR408-F/R (Table 1), and cloned into the *Xba*I/*Sac*I sites of pBI121 to generate the *Sm-MIR408*-overexpressing vector *35S::MIR408*. We amplified a 723 bp fragment from the upstream region of *Sm-MIR408* with the primers P-MIR408-F/R (Table 1) and cloned it into the *Pst*I/*Nco*I sites of pCAMBIA-1391Z to obtain the *MIR408pro::GUS* construct. These recombined vectors were then introduced into *N. benthamiana* using the *Agrobacterium*-mediated transformation method. Putative transgenic shoots were selected on agar media containing the presence of 50 mg/L kanamycin for expressing the *35S::MIR408* construct, and 25 mg/L hygromycin for expressing *MIR408pro::GUS* construct, and allowed to propagate. T2 generation transgenic tobacco were selected and analyzed.

### 4.3. Molecular Analysis of Transgenic Tobacco

To detect whether *35S::MIR408* was integrated into the genome of tobacco, the *CaMV35S* promoter was amplified by PCR with primers 35S-F/R (Table 1) and genomic DNA extracted from the leaves of kanamycin-resistant tobacco was used as templates.

Total RNA was isolated from the leaves of T2 transgenic lines and WT samples with TRIzol reagent (Invitrogen, CA, USA), and reverse-transcribed into first-strand complementary DNA (cDNA) with PrimeScript™ RT Master Mix (Takara, Dalian, China), following the manufacturer’s protocols. The resultant cDNAs were amplified with RT-*MIR408*-F/RT-*MIR408*-R primers (Table 1) to evaluate the transcript level of *Sm*-*MIR408*. qRT-PCR was performed on a Roche LightCycler^®^ 96 System by using SYBR^®^ Green Premix Ex Taq™ (TaKaRa). The *N. benthamiana* β-*actin* gene was used as the internal control, and amplified with primers *NbActin*-F/*NbActin*-R (Table 1).

To determine the level of mature miR408 in *N. benthamiana*, first-strand cDNA was synthesized using the Mir-X™ miRNA First-Strand Synthesis Kit (TaKaRa). The miRNA-specific sequences were applied as sense primers, and antisense primers were supplied by the manufacturer. U6 snRNA was used for normalization. The thermal cycling program was as follows: 95 °C for 60 s; 45 cycles of 95 °C for 10 s and 60 °C for 30 s; a cycle of 95 °C for 10 s, 65 °C for 60 s, and 97 °C for 1 s; and a cycle of 37 °C for 30 s to determine the dissociation curves of the amplified products. All reactions were performed with three biological replicates. The primers used for qRT-PCR were listed in Table 1.

### 4.4. Copper and Salt Treatment to S. miltiorrhiza and Tobacco

To analyze how *Sm-MIR408* responds to copper, the seeds of *S. miltiorrhiza* were surface-sterilized and germinated on MS medium. Two-week-old uniform *S. miltiorrhiza* seedlings were transferred to standard MS medium (0.1 μM copper), MS medium-deficient copper (0 μM, MS-Cu), and sufficient copper (5 μM, MS+Cu) for 7 days, followed by RNA isolation and RT-qPCR analysis. The seeds of transgenic tobacco expressing *MIR408pro::GUS* and WT were surface-sterilized and germinated on MS medium. One-week-old tobacco seedlings were transferred to MS, MS−Cu and MS+Cu for three weeks, followed by GUS staining.

For salt treatment, one-month-old *S. miltiorrhiza* seedlings grown in soil were watered with 150 mM NaCl for 24 h, and harvested for RT-qPCR analysis, while the control was watered with ddH_2_O. The seeds of transgenic tobacco-expressing *MIR408pro::GUS* and WT were cultured vertically on MS medium and MS medium containing 150 mM NaCl for three weeks, and followed by GUS staining.

### 4.5. GUS Bioassays

T2 generation transgenic tobacco expressing *MIR408pro::GUS* were used for analysis. The seeds were germinated on MS medium containing the presence of 25 mg/L hygromycin. One-month-old transgenic seedlings were transferred to half-strength of Hoagland salt for 2 days. These were subjected to Hoagland salts supplemented with 150 mM NaCl for 24 h. GUS histochemical staining were performed as described by Jefferson [59].

### 4.6. Histochemical Detection of H_2_O_2_ and O_2_^−^

The tobacco seedlings were incubated in NBT solution (1mg/mL in 10 mM phosphate buffer; pH 7.8) overnight in the dark until blue spots showing the accumulation of O_2_^−^ appeared. The accumulation of H_2_O_2_ was detected by immersing the plants in a 1 mg/mL solution of DAB, pH 3.8, overnight. Stained plants were soaked in 95% ethanol overnight to remove chlorophyll.

### 4.7. Determination of H_2_O_2_ Content

A 100–150 mg sample of fresh tissue was powdered in a mortar together with 1 mL of frozen acetone and a small amount of quartz sand. The homogenate was centrifuged at 8000 g for 10 min at 4 °C, and all the supernatant was taken and set on ice to be tested. The content of H_2_O_2_ was determined following the protocol of the Micro Hydrogen Peroxide Assay Kit (Solarbio, Beijing, China).

### 4.8. Measurement of SOD, POD, and CAT Activities

The seedlings were ground to powder in liquid nitrogen. We added 5 mL of 50 mmol/L phosphate-buffered saline containing 1% β-mercaptoethanol and 1% polyvinylpyrrolidone (pH 7.8) to 0.5 g of sample, and incubated this for 60 min at 4 °C After centrifugating for 20 min with 10,000 rpm at 4 °C, the supernatant (enzyme extraction) was used for analysis. The activities of POD, CAT, and SOD were measured by spectrophotometer utilizing commercial kit (A084-3, A007-1 and A001-4, Jiancheng, Nanjing, China) following the manufacturer’s instructions.

### 4.9. Expression Profile of ROS-Related Genes and Sm-MIR408 Response to Salt Stress

We performed RT-qPCR to determine the transcript level of *Sm-MIR408* in response to copper and salt treatment. The housekeeping gene *SmUbiquitin* in *S. miltiorrhiza* was used as the internal control, and amplified with primers *SmUbi*-F/*SmUbi*-R. The *N. benthamiana* β-actin gene was used an internal control to quantify the relative expression levels of *NbPOD*, *NbCAT* and *NbSOD* using RT-qPCR assay. To calculate the relative mRNA levels, raw data from RT-qPCR was analyzed using the standard curve method, normalized by the raw data of the internal control.

## 5. Conclusions

We confirmed that the expression of *Sm-MIR408* was up-regulated under salt stress and copper starvation in *S. miltiorrhiza*. Overexpression of *Sm-MIR408* in tobacco improved salt tolerance, as manifested by promoted seed germination, reduced levels of reactive oxygen species, increased activities of SOD, POD, and CAT and the expression levels of their genes. MiR408 might serve as a potential target for genetic manipulations to engineer salt stress tolerance in diverse plants.

## Figures and Tables

**Table 1:** List of primers used for PCR.

Primer	Oligo Sequence 5′ to 3′
*SmUbi*-F	ACCCTCACGGGGAAGACCATC
*SmUbi*-R	ACCACGGAGACGGAGGACAAG
*MIR408*-F	ACAGAAAATGGAGGCGAAGAAG
*MIR408*-R	GTCCCTAATCAGTGAGAGACACAGTAA
*P-MIR408*-F	ACGAGCACCGACTCTGATCATTG
*P-MIR408*-R	TCTTCTTCGCCTCCATTT TCTGTAT
35S-F	ACAAAGGCGGCAACAAACG
35S-R	GCCAGTCTTCACGGCGAGT
RT-*MIR408*-F	ACGGGGACGAGACAGAGCAT
RT-*MIR408*-R	GGCTTTCACACCAGCAACATAG
*NbActin*-F	CGTTATGGTTGGAATGGGACAGAA
*NbActin*-R	AAGAACAGGGTGCTCCTCGTGG
*Copper-transporting ATPase PAA2*-F	AAGAGGTCATCGGGGTTAGGA
*Copper-transporting ATPase PAA2*-R	GTAGCGTGAGTTCCACAGCATAAAG
*Uclacyanin*-2-F	ACTTGACGCCTCCGACCACT
*Uclacyanin*-2-R	ACTGCCTCTTCCCTAGACCATG
*NbSOD*-F	GGAGAGCCTTGTCTGATGG
*NbSOD*-R	TGGGTCCTGATTAGCAGTGGT
*NbPOD*-F	GTTGAGAGTTCTTGTCCTGGTGTT
*NbPOD*-R	TATTGGCTCCTCCCTGGTTTG
*NbCAT*-F	CACAGCCACGCTACTCAAGAC
*NbCAT*-R	CCACCCACCGACGAATAAAG
